# A 12-gene panel in estimating hormone-treatment responses of castration-resistant prostate cancer patients generated using a combined analysis of bulk and single-cell sequencing data

**DOI:** 10.1080/07853890.2023.2260387

**Published:** 2023-09-20

**Authors:** Juanlan Huang, Dale Liu, Jun Li, Jing Xu, Shaowei Dong, Hao Zhang

**Affiliations:** a Department of Health Management, Shenzhen People’s Hospital (The Second Clinical Medical College, Jinan University, Guangzhou, China; b The First Affiliated Hospital, Southern University of Science and Technology), Shenzhen, China; c Department of Urology, Shenzhen People’s Hospital (The Second Clinical Medical College, Jinan University, Guangzhou, China; d Department of Hematology and Oncology, Shenzhen Children’s Hospital of China Medical University, Shenzhen, China; e Department of Pathology, Shenzhen People’s Hospital (The Second Clinical Medical College, Jinan University, Guangzhou, China; f Department of General Surgery, The First Affiliated Hospital of Jinan University, Guangzhou, China; g Department of Thyroid, Breast and Hernia Surgery, The Second Affiliated Hospital of Shantou University Medical College, Shantou, China

**Keywords:** CRPC, hormone-treatment, 12-gene panel, combined analysis, scRNAseq data

## Abstract

**Background:**

Castration-resistant prostate cancer (CRPC) represents one type of advanced prostate cancer (PCa) with a median survival time of 1–2 years. Currently, there is a lack of reliable gene panels in predicting hormone treatment (HT) responses due to limited knowledge of CRPC-specific tumor-microenvironment (TME) characteristics.

**Methods:**

In this study, we first screened for up-regulated genes in CRPC samples using bulk-sequencing data retrieved from TCGA online database, and further investigated the expression status of these genes in four sets of downloaded single-cell RNA sequencing (scRNAseq) data: GSE117403 containing 16 normal human prostate samples; GSE141445 containing 13 PCa samples; GSE176031 containing 11 PCa samples and GSE137829 containing 6 CRPC samples.

**Results:**

We identified a series of CRPC-specific TME characteristics including an enriched number of *PEG10*^+^ neuroendocrine cells, elevated expression of *PPIB*/*CCDC74A/GAPDH/AR* genes in tumor cells, increased expression of *FAP/TGFB1* in cancer-associated fibroblasts (CAFs), suppressed immune environment featured by enhanced M2 macrophage polarization, T cell exhaustion and increased number of regulatory B cells. We further established a 12-gene panel using these characteristics and showed that this panel could separate CRPC samples from PCa samples (AUC of 0.78), and CRPC patients with higher panel scores tended to have treatment failure or progression (*R* = −0.47, *p* = 0.019).

**Conclusions:**

Based on these unique TME characteristics of CRPC, we established a prediction tool for estimating the duration of HT responses in PCa treatment. Our results suggest mechanisms by which prostate cancer becomes castrate resistant. Further study of PEG10 (and/or others) to evaluate therapeutic efficacy should be considered.

## KEY MESSAGES

Increased number of PEG10+ neuroendocrine cells is associated with recurrence of HT PCa patients

A 12-gene panel could be used to predict duration of HT responses in PCa treatment

## Introduction

Prostate cancer (PCa) remains one of the most common malignant tumors that endanger men’s health. In 2022, there are 268,490 new PCa patients in the United States (1^st^) which account for 27% of the newly diagnosed cancer cases in men [1]. Hormone therapy (HT) or androgen deprivation therapy (ADT) is the first line of treatment for PCa, however, after HT, around 10–20% of the cases would progress to a more aggressive stage: castration-resistant prostate cancer (CRPC) [2,3]. Treatment options for CRPC are largely limited, and the median survival time for men with metastatic CRPC is 1–2 years [4], hence it is necessary to establish a reliable prediction tool for CRPC progression while supporting the clinical decisions of switching therapy methods before delaying to CRPC stage.

Understanding the CRPC-specific tumor-microenvironment (TME) features is the key to developing an accurate prediction tool. Recently, with the help of single-cell RNA sequencing (scRNAseq) technology, one can elucidate the specific TME characteristics and intercellular communications in CRPC samples at a cellular level. Dong et al. [5] revealed a luminal-neuroendocrine transdifferentiation pattern in 6 human CRPC samples; Wang et al. [6] discovered a set of new features related to the divergence of neuroendocrine prostate cancer (NEPC, an advanced type of CRPC) from the original androgen-dependent PCa; Cheng et al. [7] identified a small set of CRPC-like cells in hormone-naïve early PCa samples and proved the relevance of these cells with biochemical recurrence. However, none of the current CRPC-related scRNAseq studies have involved systematic comparison of the differences between normal prostate samples, PCa samples and CRPC samples, not to mention designing prediction algorithms based on these differences.

In this study, we re-investigated TME features using 4 sets of downloaded scRNAseq data involving normal prostate samples, PCa samples, and CRPC samples, combined with bulk-sequencing data of CRPC samples from TCGA database and discovered a series of CRPC-specific features. Moreover, we established a 12-gene panel based on these features and showed that this panel had a good performance in predicting HT responses during PCa treatments.

## Materials and Methods

### Patient information

Two patients diagnosed with primary PCa and one patient diagnosed with CRPC were involved in this study. Their frozen tumor tissue sections were obtained from Pathology department of Shenzhen People’s Hospital. This study was performed in accordance with the guidelines of the 1975 Declaration of Helsinki.

### Preparation of TCGA PRAD cohort

Gene expression data (counts and FPKM: fragments per kilobase of exon per million mapped fragments) and sample phenotype data of the prostate adenocarcinoma samples (TCGA-PRAD) was retrieved from UCSC-Xena database (https://xenabrowser.net/). Normal samples were determined as ‘Solid Tissue Normal’ in ‘sample_type.sample’ term; tumor samples were determined as the rest in ‘sample_type.sample’; samples with recurrence were determined as tumor samples with ‘YES’ in ‘new_tumor_event_after_initial_treatment’ term; samples without recurrence were determined as tumor samples with ‘NO’ in ‘new_tumor_event_after_initial_treatment’ term; HT_YES samples were determined as recurrent samples with ‘Hormone Therapy’ in ‘therapy_type’ (onset of castration resistance status after initial hormone treatment), and HT_NO samples were determined as non-recurrent samples with ‘Hormone Therapy’ in ‘therapy_type’.

### Identification of differentially expressed genes

To screen for differentially expressed genes, we applied Deseq2 (R package) to raw-count reads of HT_YES and HT_NO samples. Log2FC of 1 and p.adjust value of 0.05 were used as significance cutoff.

### scRNAseq analysis of four datasets

Count matrix files were downloaded from GEO database (www.ncbi.nlm.nih.gov/geo/) under their accession numbers (GSE117403 containing expression profiles of 105,208 cells from 16 normal human prostate samples [8]; GSE141445 containing expression profiles of 31,402 cells from 13 PCa patients [9]; GSE176031 containing expression profiles of 17,144 cells from 11 PCa patients [10]; GSE137829 containing expression profiles of 20,174 cells from 6 CRPC patients^5^]. For each dataset, a SeuratObject was first constructed for each sample/patient using R package (Version 3.1.2) [11], followed by a doublet removal step using DoubletFinder R package [12] (10% double formation rate). Samples from each dataset were integrated using ‘FindIntegrationAnchors’ and ‘IntegrateData’. For the integrated object, a ‘ScaleData’ step was first applied, followed by a ‘RunPCA’ step, ‘FindNeighbors’ step, and a ‘FindClusters’ step. The clusters were visualized using UMAP (Uniform Manifold Approximation and Projection) method.

### Similarity analysis using Pearson correlation method

Data normalization among different datasets was performed using log2(CPM + 1), and mean expression values for all genes were calculated for each subgroup. Pearson correlation value between two subgroups was calculated using R cor.test() function and the correlation results between all subgroup combinations were visualized using R ‘pheatmap’ package.

### TCGA and CPGEA module score calculation

Lists of TCGA and CPGEA module genes were retrieved from Wang et al. [6]. For each involved gene, a zscore transformation was performed to log2(CPM + 1) normalized matrix, and module score for each cell was calculated as sums of zscore values of all involved genes. Module scores between cells from different subgroups were compared using a student t-test and a p.value of 0.05 was used as significance cutoff.

### Identification of subgroup-featured genes

Subgroup-featured genes were determined as genes with the highest expression values across all subgroups. Gene expression values were first normalized using log2(CPM + 1), and for a given gene A, if its expression values were significantly higher in cells from subgroup B (dataset C) compared to the values from other subgroups (significance was determined using R Limma-trend package), and gene A was expressed in over 20% of the cells from subgroup B, gene A was considered as one of the B subgroup-featured genes. KEGG enrichment analysis of subgroup-featured genes was performed using ‘clusterProfiler’ R package (p.adjust< 0.05), and top enriched KEGG terms were selected and displayed.

### Patient information

The CPRC patient involved in the validation process of this study was enrolled in Shenzhen People’s Hospital. This CRPC patient had a laparoscopic radical prostatectomy in the year 2018, followed by three rounds of endocrine therapy (Bicalutamide: 50 mg; Goserelin: 10.8 mg), and the progression happened in the year 2022. This is a luminal CRPC based on the diagnosis.

## Immunostaining

Tumor tissue sections from two PCa patients and one CRPC patient were obtained from the pathology department of Shenzhen People’s Hospital. Prior to antibody incubation, all sections were washed in 3% H_2_O_2_ at room temperature for 10 min. The following two primary antibodies were used in this study: anti-EPCAM (1:500, abcam, ab223582) and anti-PEG10 (1:500, proteintech, 14412-1-AP). After being rinsed with 1X PBS at room temperature for 3 times, the sections were first incubated with primary antibodies for 2h at room temperature, followed by 3 times of 1X PBS rinsing, 1h of incubation with goat anti-rabbit H&L Cy5 secondary antibody (Abcam ab6564), and 3 more times of 1X PBS rinsing. The following stains (green: Alexa Fluor^TM^ 488 Tyramide, Invitrogen B40922; red: Alexa Fluor^TM^ 555 Tyramide, Invitrogen B40923) were used in the final staining step.

### Ligand-Receptor (L-R) intensity calculation

LR intensities between different subgroups in GSE137829 dataset were calculated as follows: 1) Location information of all proteins was retrieved from THPA (The Human Protein Atlas, www.proteinatlas.org) website, in which secreted proteins were determined as ‘Secreted’ in ‘Predicted location’ term, and membrane proteins were determined as ‘Plasma membrane’ in ‘Predicted location’ term; 2) Protein-Protein interaction information was retrieved from BioGRID (www.thebiogrid.org) database [13]; 3) Intensity between subgroup A (Ligand) and subgroup B (Receptor) was added up by the intensity of each LR pair calculated by multiply of mean expression value of ligand (secreted protein from subgroup A) and mean expression value of receptor (membrane protein from subgroup B). P value was performed using method described in Ji et al. [14]. A randomized process was performed to all cells for 1000 times, and the intensity was compared to these randomized intensities. The ranks of target intensities were used as statistical values.

### Receiver operating characteristic (ROC) analysis

ROC analysis was performed using R package ‘ROCR’ and ‘pROC’, and the AUC (area under curves) values were calculated using auc() function.

## Results

### Identification of differentially expressed genes in TCGA CRPC samples

**Figure 1.**
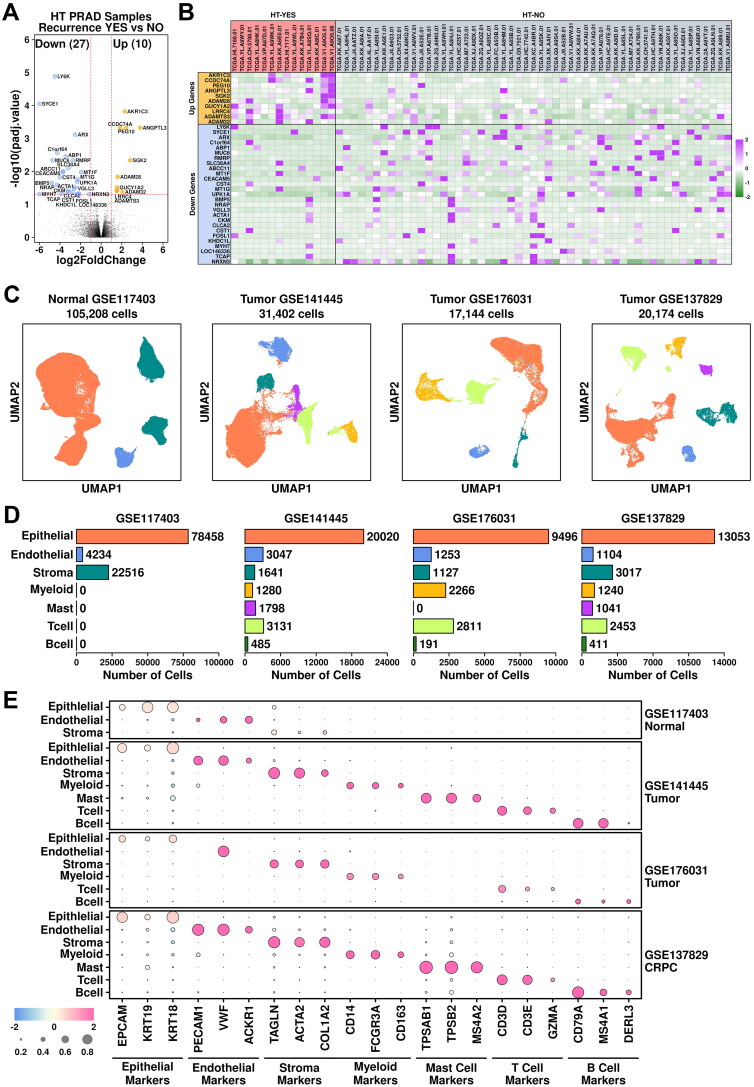
Combined analysis of bulk-sequencing data and scRNAseq data. (A) Volcano plot representing the identification of differentially expressed genes in HT-YES (hormone-treated PCa patients with recurrence) samples vs. HT-NO (hormone-treated PCa patients without recurrence). Up-regulated genes (p.adj < 0.05, log2fc > 1) are labeled in orange and down-regulated genes (p.adj < 0.05, log2fc < -1) are labeled in blue. (B) Expression status of up-regulated and down-regulated genes across HT-YES and HT-NO samples. (C) Uniform Manifold Approximation and Projection (UMAP) plot showing the distribution patterns of subgroups in each dataset. (D) Bar plot showing the number of cells in each subgroup from each dataset. (E) Dot plot representing the relative expression level (color) and the ratio of gene-expressing cells (size) of all marker genes used in the definition of each subgroup.

To screen for differentially expressed genes in CRPC patients, we first retrieved gene expression data of prostate adenocarcinoma samples from The Cancer Genome Atlas (TCGA-PRAD). Two sets of samples were selected: PRAD samples with recurrence after hormone treatment (HT-YES, 14 samples) and PRAD samples without recurrence after hormone treatment (HT-NO, 53 samples). Detailed sample information is listed in **Supplementary Table 1**. A further transcriptome-wide screening of differentially expressed genes was performed using DESeq2 [15], and the results are illustrated in Figure 1A. 10 up-regulated genes (log2FC > 1 & P.adjust < 0.05) and 27 down-regulated genes (log2FC < −1 & P.adjust < 0.05) are identified (listed in **Supplementary Table 4**), and their relative expression values across these HT-YES/HT-NO samples are listed in Figure 1B.

### Construction of single-cell transcriptome atlas

To investigate CPRC-specific TME features from single-cell levels, we retrieved four scRNAseq datasets from GEO database: GSE117403 containing expression profiles of 105,208 cells from 16 normal human prostate samples [8]; GSE141445 containing expression profiles of 31,402 cells from 13 PCa patients [9]; GSE176031 containing expression profiles of 17,144 cells from 11 PCa patients [10]; GSE137829 containing expression profiles of 20,174 cells from 6 CRPC patients [5].

Due to the disturbance of batch effects across these four datasets (10x Genomics platform used in GSE117403/GSE141445/GSE137829; Seq-Well platform used in GSE176031), we performed scRNAseq analysis separately to these four datasets instead of the integrating them together. To compare gene expression levels across these four datasets, a log2(CPM + 1) normalization was first performed to all involved cells, followed by a transcriptome-level comparison using Limma-trend method [16,17]. All the expression-level analysis was performed using this method.

The following standard seRNAseq analysis procedures were applied: a matrix reconstruction step using seurat; duplet removal step using DoubletFinder [12]; an integration/reduction step using standard seurat protocol. Clusters from all four datasets were visualized using UMAP method, as illustrated in Figure 1C. Epithelial cells (78,458 cells in GSE117403, 66.83%; 20,020 cells in GSE141445, 63.75%; 9,496 cells in GSE176031, 55.39%; 13,053 cells in GSE137829, 64.70%) are marked using *EPCAM/KRT19/KRT18*; Endothelial cells (4,234 cells in GSE117403, 4.02%; 3,047 cells in GSE141445, 9.70%; 1,253 cells in GSE176031, 7.30%; 1,104 cells in GSE137829, 5.47%) are marked using *PECAM1/VWF/ACKR1*; Stroma cells (22,516 cells in GSE117403, 21.40%; 1,641 cells in GSE141445, 5.22%; 1,127 cells in GSE176031, 6.57%; 3,017 cells in GSE137829, 14.95%) are marked using *TAGLN/ACTA2/COL1A2*; Myeloid cells (1,280 cells in GSE141445, 4.08%; 2,266 cells in GSE176031, 13.22%; 1,240 cells in GSE137829, 6.15%) are marked using *CD14/FCGR3A/CD163*; Mast cells (1,798 cells in GSE141445, 5.72%; 1,041 cells in GSE137829, 5.15%) are marked using *TPSAB1/TPSB2/MS4A2*; T cells (3,131 cells in GSE141445, 9.97%; 2,811 cells in GSE176031, 16.40%; 2,453 cells in GSE137829, 12.16%) are marked using *CD3D/CD3E/GZMA*; B cells (485 cells in GSE141445, 1.56%; 191 cells in GSE176031, 1.11%; 411 cells in GSE137829, 2.04%) are marked using *CD79A/MS4A1/DERL3*, as summarized in Figure 1C,D.

### Heterogeneity of cancerous cells in tumor and CRPC samples

**Figure 2.**
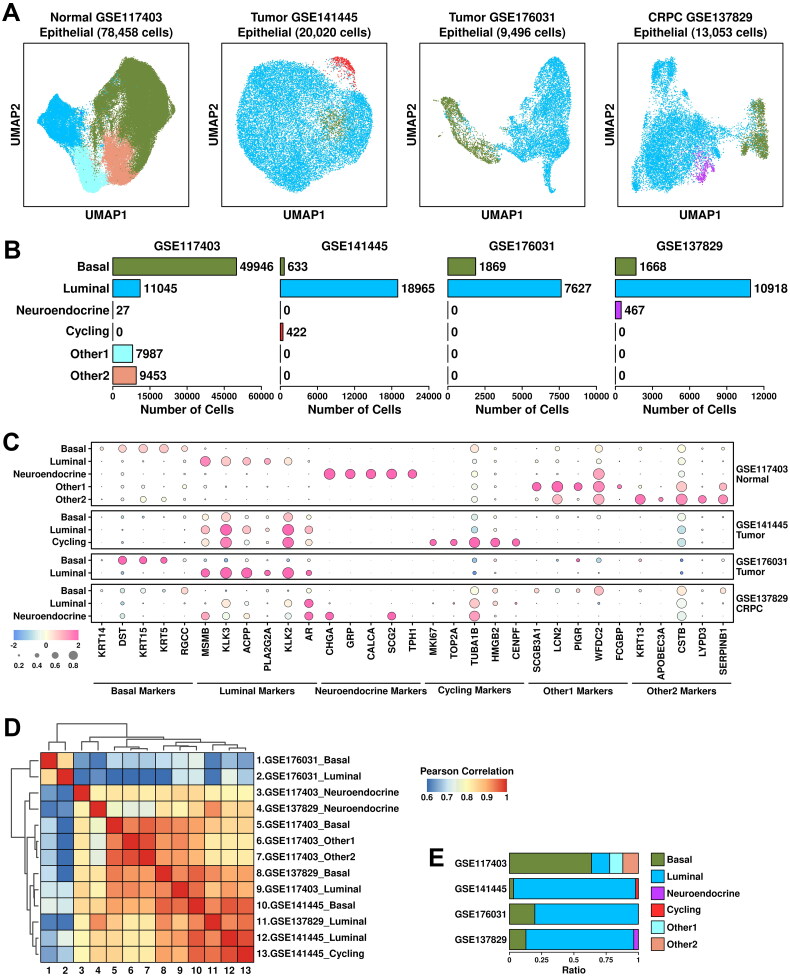
Epithelial component analysis across four datasets. (A) UMAP plot showing the distribution patterns of subgroups in each dataset. (B) Bar plot showing the number of cells in each subgroup from each dataset. (C) Dot plot representing the relative expression level (color) and the ratio of gene-expressing cells (size) of all marker genes used in the definition of each subgroup. (D) Heatmap representing the similarity of expression patterns among different subgroups. The expression pattern is defined as the mean expression values of all genes within one subgroup, and a Pearson correlation analysis is performed to compare the similarity. (E) Bar plot representing the relative ratio of each epithelial subgroup across four datasets.

Epithelial cells account for around 60% of the cells among all four datasets. To explore CRPC-specific features in epithelial cells, we further clustered these cells into basal cells (marked by *KRT14/DST/KRT15/KRT5/RGCC*), luminal cells (marked by *MSMB/KLK3/ACPP/PLA2G2A/KLK2*), neuroendocrine cells (marked by *CHGA/GRP/CALCA/SCG2/TPH1*), cycling cells (marked by *MKI67/TOP2A/TUBA1B/HMGB2/CENPF*), other1 cells (subgroup defined in the original publication, marked by *SCGB3A1/LCN2/PIGR/WFDC2/FCGBP*) and other2 cells (subgroup defined in the original publication, marked by *KRT13/APOBEC3A/CSTB/LYPD3/SERPINB1*) (Figure 2A). Among epithelial cells from GSE117403 dataset, there are 49,946 basal cells (63.66%), 11,045 luminal cells (14.08%), 27 neuroendocrine cells (0.034%), 7,987 other1 cells (10.18%) and 9,453 other2 cells (12.05%); among epithelial cells from GSE141445 dataset, there are 633 basal cells (3.16%), 18,965 luminal cells (94.73%) and 422 cycling cells (2.11%); among epithelial cells from GSE176031 dataset, there are 1,869 basal cells (19.65%) and 7,627 luminal cells (80.32%); among epithelial cells from GSE137829 dataset, there are 1,668 basal cells (12.78%), 10,918 luminal cells (83.64%) and 467 neuroendocrine cells (3.58%), respectively, as detailed in Figure 2B. The relative expression status of involved markers in defining each subgroup is shown in Figure 2C.

We performed inferCNV analysis to all epithelial cells from these four datasets using expression profiles of t cells (from GSE141445, GSE176031 and GSE137829 datasets), and the results are listed in Supplementary Figure 1. Compared to epithelial cells in normal prostate samples (GSE117403), more copy number variance features were found in two tumor samples (GSE141445, GSE176031) and CRPC samples (GSE137829).

Similarity among subgroups from each dataset was evaluated using Pearson correlation analysis (detailed in Materials and Methods). The results are summarized in Figure 2D. Basal and luminal subgroups from GSE176031 dataset (tumor) are clustered in a separate branch, suggesting that cells from this dataset are more different than cells from other datasets. This might be possibly due to different platforms during library construction; Luminal and cycling cells from GSE141445 dataset (tumor) are clustered together with luminal cells from GSE137829 dataset (CRPC), suggesting the intrinsic similarity among these subgroups. There is a huge increase in luminal cell ratio from PCa and CRPC samples compared to this from normal samples (Figure 2E), suggesting that luminal cells are the main cancerous cells in these tumor/CRPC samples.

**Figure 3.**
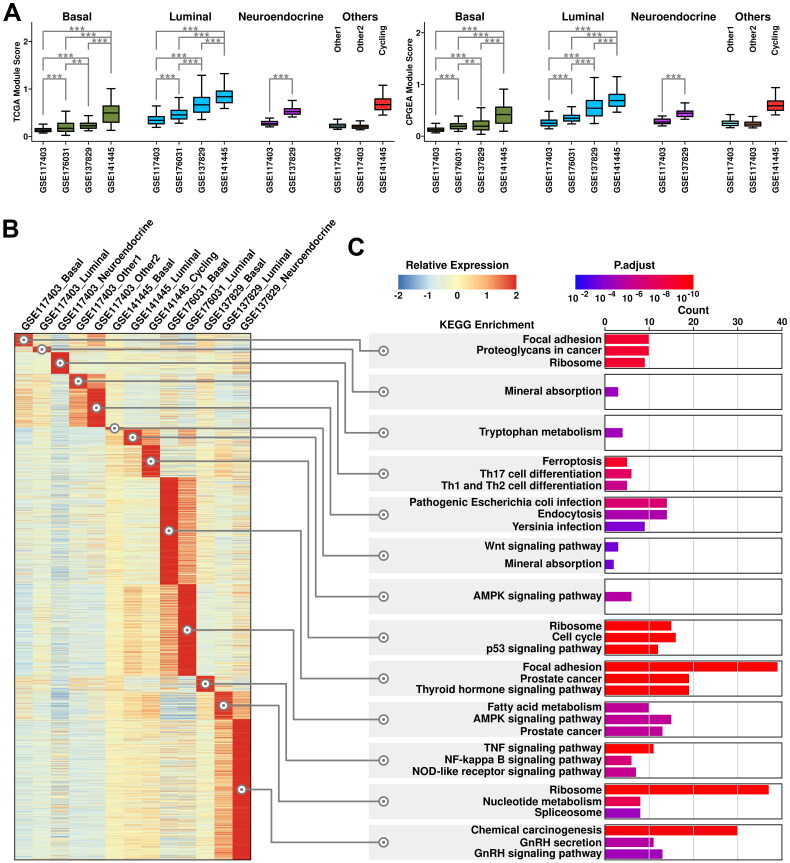
Heterogeneity of cancerous cells across all 4 datasets. (A) Expression status of TCGA module and CPGEA module across all subgroups. A student t.test is performed for each comparison (*, *p* < 0.05; **, *p* < 0.01; ***, *p* < 0.001). (B) Expression of subgroup-featured genes across different subgroups. (C) List of top enriched KEGG terms of featured genes from each subgroup.

To investigate the tumor-associated features of these subgroups, we applied two PCA-related gene panels [6] to these datasets and the results are shown in Figure 3A. TCGA module is derived from TCGA-PRAD dataset which includes 199 upregulated genes; CPGEA module is derived from CPGEA (Chinese Prostate Cancer Genome and Epigenome Atlas) database which includes 248 upregulated genes (listed in **Supplementary Table 2**). Luminal subgroups have relatively higher TCGA/CPGEA module scores compared to those in basal/neuroendocrine/others subgroups; tumor/CRPC subgroups have relatively higher module scores compared to those in normal subgroups. These results further support the robustness of the analysis process.

Subgroup-featured genes (genes with the highest expression values among all the subgroups as calculated using Limma-trend, detailed in Materials and Methods) are further summarized (illustrated in Figure 3B) and their KEGG enrichment results are listed in Figure 3C. Interestingly, featured genes in neuroendocrine cells from CRPC samples are enriched in KEGG pathways including GnRH (Gonadotropin-releasing hormone) secretion and GnRH signaling pathways. Moreover, these enrichments are not seen in neuroendocrine cells from normal samples, indicating more GnRH production might be related to CRPC progression.

### Generation of a 6-gene panel using CRPC-specific epithelial features

**Figure 4.**
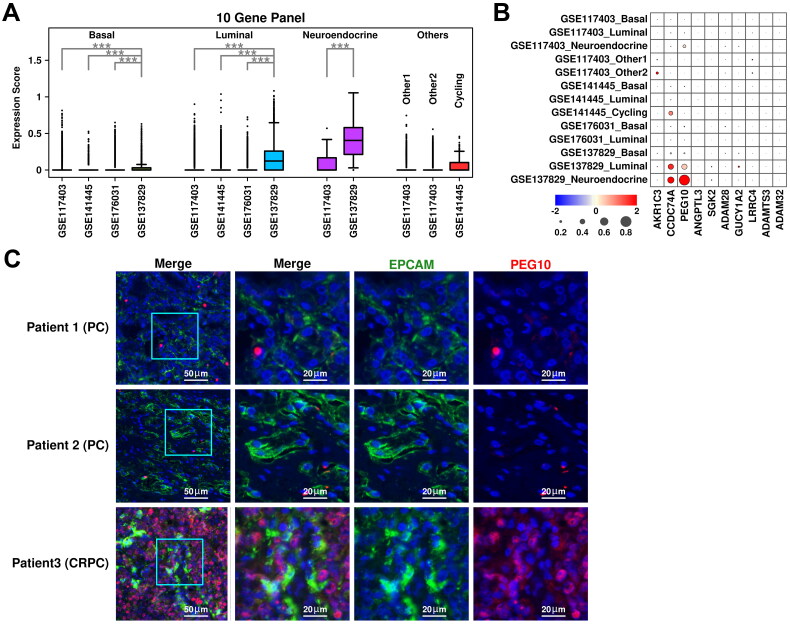
Cross-validation of CRPC-featured genes. (A) Expression status of the 10 up-regulated genes across different subgroups. A student t.test is performed for each comparison (*, *p* < 0.05; **, *p* < 0.01; ***, *p* < 0.001). (B) Dot plot representing the relative expression level (color) and the ratio of gene-expressing cells (size) of 10 up-regulated genes in each subgroup. (C) Immunostaining results showing anti-EPCAM (green) and anti-PEG10 (red) labeled PCa tissues (Patient 1 and Patient 2) and CRPC tissue (Patient 3).

The expression status of the top 10 upregulated genes in HT-YES samples is also examined among epithelial subgroups across all 4 datasets. Neuroendocrine subgroup from CRPC samples has the highest expression scores of these genes, followed by luminal subgroup from CRPC samples, as illustrated in Figure 4A. Specifically, both *PEG10* (Paternally expressed 10) and *CCDC74A* (Coiled-coil domain containing 74 A) have higher expression values in neuroendocrine/luminal subgroups from CRPC samples compared to those from other subgroups (Figure 4B). To further verify the expression status of *PEG10* in CRPC epithelial cells, we labeled PCA tissues (Patient 1 and Patient 2) and CRPC tissue (Patient 3) with anti-EPCAM (green) and anti-PEG10 (red) antibodies, and the immunostaining results are shown in Figure 4C. More *PEG10*-expressing epithelial cells (green + red) are examined in CRPC tissue compared to these in PCa tissues, which further supports our scRNAseq results.

**Figure 5.**
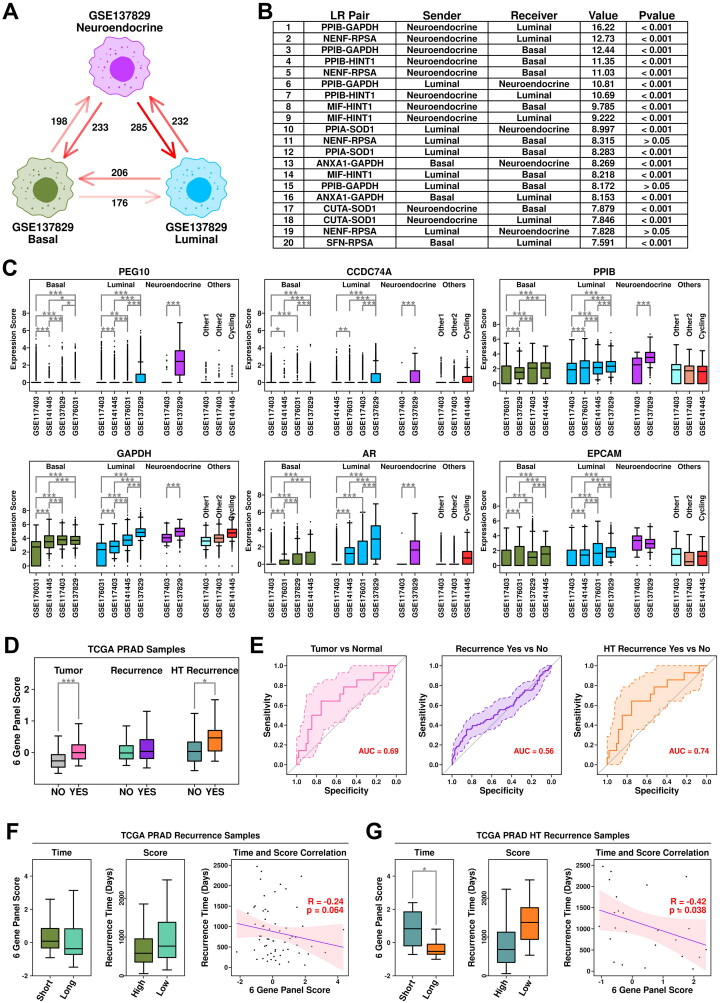
Generation of a 6-gene panel using CRPC-specific epithelial features. (A) Cartoon plot representing the communication intensity between different epithelial subgroups in CRPC samples. (B) List of top 20 LR pairs between different epithelial subgroups in CRPC samples. (C) Expression status of *PEG10*, *CCDC74A*, *PPIB*, *GAPDH*, *AR,* and *EPCAM* across different subgroups. A student t.test is performed for each comparison (*, *p* < 0.05; **, *p* < 0.01; ***, *p* < 0.001). (D) Expression scores of 6-gene panel across different samples in TCGA PRAD cohort. A student t.test is performed for each comparison (*, *p* < 0.05; **, *p* < 0.01; ***, *p* < 0.001). (E) ROC plots representing the performance of the 6-gene panel in separating normal samples from tumor samples, recurrent tumor samples from non-recurrent tumor samples and HT recurrent samples from HT non-recurrent samples. (F) For recurrent tumor samples, left plot: samples with shorter recurrence time (time < = median) have higher (t.test not significant) 6-gene panel scores than samples with longer recurrence time (time > median); middle plot: samples with higher 6-gene panel scores (score > = median) have shorter (t.test not significant) recurrence time than samples with lower 6-gene panel scores (score < median); right plot: pearson correlation between recurrence time and 6-gene panel scores in recurrent tumor samples. (G) For recurrent HT samples, left plot: samples with shorter recurrence time (time < = median) have higher (t.test significant, *, *p* < 0.05; **, *p* < 0.01; ***, *p* < 0.001) 6-gene panel scores than samples with longer recurrence time (time > median); middle plot: samples with higher 6-gene panel scores (score > = median) have shorter (t.test not significant) recurrence time than samples with lower 6-gene panel scores (score < median); right plot: pearson correlation between recurrence time and 6-gene panel scores in recurrent tumor samples.

Secreted ligand is one of the most direct methods that target cells use to reshape their microenvironments. To investigate the communication status among CRPC epithelial subgroups, we calculated Ligand-Receptor (L-R) intensity between different subgroups, and the results are summarized in Figure 5A. The LR intensity between neuroendocrine cells (Secreted Ligands) and luminal cells (Membrane Receptors) is the strongest among all subgroup combinations, suggesting the unusually frequent communications between these two subgroups. Top-ranked L-R interactions are listed in Figure 5B. Among them, *PPIB* (Peptidylprolyl isomerase B, secreted from neuroendocrine cells) - *GAPDH* (Glyceraldehyde-3-phosphate dehydrogenase, receptors from luminal cells) ranks 1^st^ among all the L-R interactions, indicating that this interaction might play an important role in CRPC progression.

As demonstrated previously, *PEG10/CCDC74A/PPIB/GAPDH* have higher expression values in neuroendocrine and luminal subgroups from CRPC samples (Figure 5C**)**. Moreover, we also find that *AR* (Androgen receptor), a transcription factor that could be regulated by *GAPDH* [18], has the highest expression values in luminal cells from CRPC samples (Figure 5C). This *PPIB-GAPDH-AR* combination might be a key regulator in CRPC progression. Together with *EPCAM* (epithelial marker), we established a 6-gene panel containing *PEG10/CCDC74A/PPIB/GAPDH/AR/EPCAM*, and further examined the performance of this panel in the TCGA cohort, as shown in Figure 5D. Among TCGA PRAD samples, this 6-gene panel has the highest expression scores in HT samples with recurrence; specifically, the expression score is significantly higher in tumor samples compared to these in normal samples (pvalue: 3.54e-08); significantly higher in PRAD samples with recurrence compared to these in PRAD samples without recurrence (pvalue: 0.028); significantly higher in HT samples with recurrence compared to these in HT samples without recurrence (pvalue: 0.043). Regarding ROC analysis, this 6-gene panel has an auc (area under curve) score of 0.69 in separating tumor samples from normal samples, 0.56 in separating PRAD samples with recurrence from PRAD samples without recurrence, 0.74 in separating HT samples with recurrence from HT samples without recurrence (Figure 5E).

Specifically, we further explored the relationship between expression scores of this 6-gene panel and recurrence time. Among tumor samples with recurrence, samples with shorter (time < = median) recurrence time have higher panel scores compared to samples with longer (time > median) recurrence time (Short mean/median: 0.32/0.082; Long mean/median: 0.12/-0.37; pvalue: 0.52); samples with higher panel scores (score > = median) have shorter recurrence time compared to these with lower (score < median) panel scores (Higher mean/median: 754.57/582 days; Lower mean/median: 966.71/766 days; pvalue: 0.19). Pearson correlation analysis between 6-gene panel scores and recurrence time reveals a weak (not significant) negative correlation (-0.24, *p* = 0.064), as shown in Figure 5F. Among HT samples with recurrence, samples with shorter (time < = median) recurrence time have significantly higher panel scores compared to those with longer (time > median) recurrence time (Short mean/median: 0.90/0.83; Long mean/median: −0.17/-0.53; pvalue: 0.015); samples with higher panel scores (score > = median) have shorter recurrence time compared to these with lower (score < median) panel scores (Higher mean/median: 866.67/679 days; Lower mean/median: 1314.08/1376 days; pvalue: 0.14). Pearson correlation analysis between 6-gene panel scores and recurrence time reveals a significant negative correlation (-0.42, *p* = 0.038), as shown in Figure 5G. All these results suggest that this 6-gene panel has a good performance in this HT-TCGA-PRAD cohort.

### Other CRPC-specific TME features revealed in scRNAseq analysis

**Figure 6.**
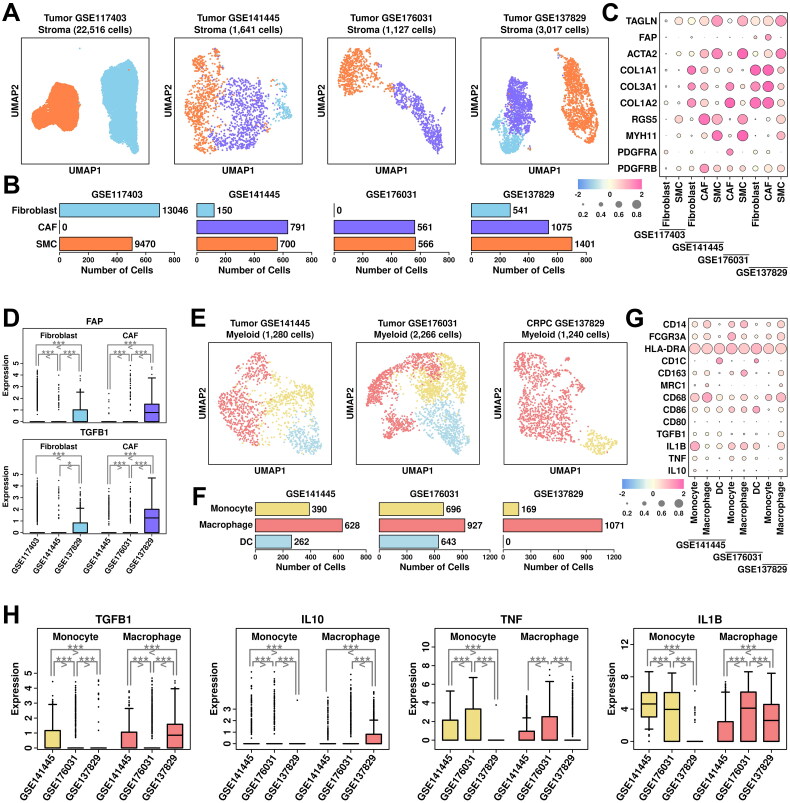
Stroma and myeloid analysis across four datasets. (A) UMAP plot showing the distribution patterns of stroma subgroups in each dataset. (B) Bar plot showing the number of cells in each stroma subgroup from each dataset. (C) Dot plot representing the relative expression level (color) and the ratio of gene-expressing cells (size) of all marker genes used in the definition of each stroma subgroup. (D) Gene expression levels of *FAP* and *TGFB1* across stroma subgroups. A student t.test is performed for each comparison (*, *p* < 0.05; **, *p* < 0.01; ***, *p* < 0.001). (E) UMAP plot showing the distribution patterns of myeloid subgroups in each dataset. (F) Bar plot showing the number of cells in each myeloid subgroup from each dataset. (G) Dot plot representing the relative expression level (color) and the ratio of gene-expressing cells (size) of all marker genes used in the definition of each myeloid subgroup. (H) Gene expression levels of *TGFB1, IL10, TNF,* and *IL1B* across myeloid subgroups. A student t.test is performed for each comparison (*, *p* < 0.05; **, *p* < 0.01; ***, *p* < 0.001).

Stroma cells were clustered into fibroblasts (marked by *COL1A1/COL3A1/COL1A2/PDGFRA*), CAF cells (marked by *FAP/ACTA2/PDGFRB*), and SMC (smooth muscle cells, marked by *TAGLN/ACTA2/RGS5/MYH11*) (Figure 6A). Among stroma cells from GSE117403 dataset, there are 13,046 fibroblast cells (57.94%), and 9,470 SMC cells (42.06%); among stroma cells from GSE141445 dataset, there are 150 fibroblast cells (9.14%), 791 CAF cells (48.20%) and 700 SMC cells (42.66%); among stroma cells from GSE176031 dataset, there are 561 CAF cells (49.78%) and 566 SMC cells (50.22%); among stroma cells from GSE137829 dataset, there are 541 fibroblast cells (17.93%), 1,075 CAF cells (35.63%) and 1,401 SMC cells (46.44%), respectively, as shown in Figure 6B. The relative expression status of involved markers in defining each subgroup is shown in Figure 6C. CAF cells play important roles in PCa progression and recurrence [19], and the expression status of two representative CAF markers, *FAP* and *TGFB1*, are summarized in Figure 6D. Among fibroblast subgroups, *FAP* expression is higher in cells from CRPC samples (GSE137829) and PCa samples (GSE141445); among CAF subgroups, both *FAP* and *TGFB1* expressions are higher in cells from CRPC samples (GSE137829) compared to cells from PCa samples (GSE141445 and GSE176031), suggesting an enhanced CAF conversion process in CRPC samples.

Myeloid cells were clustered into monocytes (marked by *CD14/FCGR3A/HLA-DRA*), macrophages (marked by *CD163/MRC1/CD68/CD86*), and dendritic cells (DC, marked by *HLA-DRA/CD1C*) (Figure 6E). Among myeloid cells from GSE141445 dataset, there are 390 monocytes (30.47%), 628 macrophages (49.06%) and 262 DC cells (20.47%); among myeloid cells from GSE176031 dataset, there are 696 monocytes (30.71%), 927 macrophages (40.91%) and 643 DC cells (28.38%); among myeloid cells from GSE137829 dataset, there are 169 monocytes (13.63%) and 1,071 macrophages (86.37%), respectively, as shown in Figure 6F. The relative expression status of involved markers in defining each subgroup is shown in Figure 6G. In macrophage cells from CRPC samples, we find relatively higher expression of M2 polarization markers *TGFB1/IL10*, as well as lower expression of M1 polarization markers *TNF/IL1B* compared to cells from PCa samples (Figure 6H), suggesting an enhanced M2 polarization process in CRPC samples.

**Figure 7.**
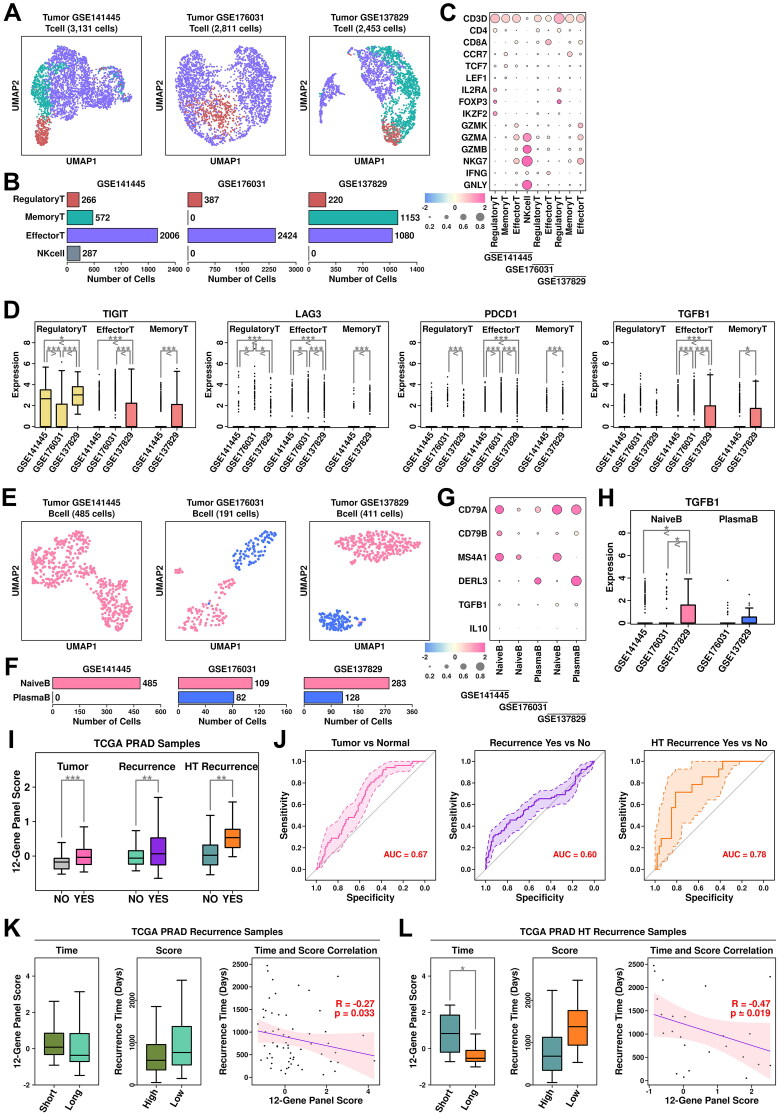
Tcell/Bcell analysis across four datasets and generation of a 12-gene panel. (A) UMAP plot showing the distribution patterns of Tcell subgroups in each dataset. (B) Bar plot showing the number of cells in each Tcell subgroup from each dataset. (C) Dot plot representing the relative expression level (color) and the ratio of gene-expressing cells (size) of all marker genes used in the definition of each Tcell subgroup. (D) Gene expression levels of *TIGIT, LAG3, PDCD1,* and *TGFB1* across stroma subgroups. A student t.test is performed to each comparison (*, *p* < 0.05; **, *p* < 0.01; ***, *p* < 0.001). (E) UMAP plot showing the distribution patterns of Bcell subgroups in each dataset. (F) Bar plot showing the number of cells in each Bcell subgroup from each dataset. (G) Dot plot representing the relative expression level (color) and the ratio of gene-expressing cells (size) of all marker genes used in the definition of each Bcell subgroup. (H) Gene expression levels of *TGFB1* across Bcell subgroups. A student t.test is performed for each comparison (*, *p* < 0.05; **, *p* < 0.01; ***, *p* < 0.001). (I) Expression scores of 12-gene panel across different samples in TCGA PRAD cohort. A student t.test is performed for each comparison (*, *p* < 0.05; **, *p* < 0.01; ***, *p* < 0.001). (J) ROC plots representing the performance of the 12-gene panel in separating normal samples from tumor samples, recurrent tumor samples from non-recurrent tumor samples and HT recurrent samples from HT non-recurrent samples. (K) For recurrent tumor samples, left plot: samples with shorter recurrence time (time < = median) have higher (t.test not significant) 121-gene panel scores than samples with longer recurrence time (time > median); middle plot: samples with higher 12-gene panel scores (score > = median) have shorter (t.test not significant) recurrence time than samples with lower 12-gene panel scores (score < median); right plot: pearson correlation between recurrence time and 6-gene panel scores in recurrent tumor samples. (L) For recurrent HT samples, left plot: samples with shorter recurrence time (time < = median) have higher (t.test significant, *, *p* < 0.05; **, *p* < 0.01; ***, *p* < 0.001) 12-gene panel scores than samples with longer recurrence time (time > median); middle plot: samples with higher 12-gene panel scores (score > = median) have shorter (t.test not significant) recurrence time than samples with lower 12-gene panel scores (score < median); right plot: pearson correlation between recurrence time and 12-gene panel scores in recurrent tumor samples.

T cells were clustered into regulatory T cells (marked by *IL2RA/FOXP3/IKZF2/CD4*), effector T cells (marked by *GZMK/GZMA/GZMB*), memory T cells (marked by *CCR7/TCF7/LEF1*), and natural killer (NK) cells (marked by *CD3D^-^/NKG7/GNLY*) (Figure 7A). Among T cells from GSE141445 dataset, there are 266 regulatory T cells (8.50%), 572 memory T cells (18.27%), 2,006 effector T cells (64.07%) and 287 NK cells (9.17%); among T cells from GSE176031 dataset, there are 387 regulatory T cells (13.77%) and 2,424 effector T cells from GSE176031 dataset; among T cells from GSE137829 dataset, there are 220 regulatory T cells (8.97%), 1,153 memory T cells (47.00%) and 1,080 effector T cells (44.03%), respectively, as shown in Figure 7B. The relative expression status of involved markers in defining each subgroup is shown in Figure 7C. In T cells from CRPC samples, we find relatively higher expression of immune checkpoint proteins including *TIGIT*, *LAG3* and *PDCD1* compared to those from PCa samples (Figure 7D). The expression of TGFB1 is also higher in T cells from CRPC samples compared to these from PCa samples. All these suggest a more severe Tcell exhaustion process in CRPC samples, leading to further immune suppression of the tumor microenvironment.

B cells were clustered into naïve B cells (marked by *CD79A/CD79B/MS4A1*) and plasma B cells (marked by *CD79A/DERL3*) (Figure 7E). Among B cells from GSE141445 dataset, all cells are defined as naïve B cells; among B cells from GSE176031, there are 109 naïve B cells (57.07%) and 82 plasma B cells (42.93%); among B cells from GSE137829, there are 283 naïve B cells (68.86%) and 128 plasma B cells (31.14%), respectively, as shown in Figure 7F. The relative expression status of involved markers in defining each subgroup is shown in Figure 7G. Regulatory B cells are one type of immune-suppressive B cells marked by high expression of *TGFB1* and *IL10*. In CRPC samples, we find higher expression of *TGFB1* in naïve B cells compared to these in PCa samples, suggesting more regulatory B cells in CRPC samples, which might also contribute to an immune-suppressive microenvironment.

### Generation of an updated 12-gene panel using CRPC-specific TME features

We added 6 more genes identified from CRPC-specific TME features (*FAP/IL10/TGFB1/TIGIT/LAG3/PDCD1*) to the original 6-gene panel and further explored the performance of this panel in TCGA cohort. Among TCGA PRAD samples, this 12-gene panel has the highest expression scores in HT tumor samples with recurrence, which is consistent with the previous 6-gene panel; specifically, the expression score is significantly higher in tumor samples compared to these in normal samples (pvalue: 3.54e-08); significantly higher in PCa samples with recurrence compared to these in PCa samples without recurrence (pvalue: 7.1e-04); significantly higher in HT samples with recurrence compared to these in HT samples without recurrence (pvalue: 6.6e-03), as shown in Figure 7I. Compared to the 6-gene panel, this 12-gene panel has slightly better discriminating ability in separating HT samples with recurrence from those without recurrence (auc: 0.78 vs. 0.74), as shown in Figure 7J.

Regarding correlations with recurrence time, compared to the 6-gene panel, there is a slightly better negative correlation between panel scores and recurrence times in PCa samples with recurrence (Figure 7K, R: −0.27 vs. −0.24) and HT samples with recurrence (Figure 7L, R: −0.47 vs. −0.42), suggesting a slightly better performance of this 12-gene panel in estimating the outcomes of HT PCa patients.

## Discussion

In this study, through a combined analysis of bulk-sequencing data and 4 sets of scRNAseq data, we successfully identified a series of CRPC-specific features such as increased number of *PEG10^+^* neuroendocrine cells, enhanced expression of PPIB/GAPDH/AR gene set in tumor cells, elevated expression of *TGFB1* in TMEs suppressed immune environments such as M2 macrophage polarization, T cell exhaustion, as well as increased number of regulatory B cells. Moreover, we established a 12-gene panel using these CRPC-specific features screened in this study, and found that this panel had a good performance in estimating the duration of HT responses in PCa patients (auc = 0.78).

The involvement of placental gene *PEG10* in neuroendocrine prostate cancer (NEPC, one type of highly aggressive variant of CRPC) have been reported in many studies. Kim et al. [20] showed that *PEG10* is associated with neuroendocrine differentiation after AR axis-directed therapy, and Akamatsu et al. [21] reported that *PEG10* could promote the progression of NEPC through stimulating cell-cycle progression *via RB1* and *TP53* loss and regulating Snail expression *via* TGF-beta signaling. In this study, we found that *PEG10* is up-regulated in both HT PCa samples with recurrence and neuroendocrine cells from CRPC samples, suggesting that PEG10 is a promising target in CRPC exploration.

AR-signaling axis is crucial in all stages of prostate cancer, and the involvement of *AR* in CRPC progression has been observed for a long time. Visakorpi et al. [22] observed that over 30% of CRPC patients have AR gene amplification; Chen et al. [23] reported that increased expression of AR gene was sufficient to accelerate the CRPC process. Generally, it is considered that AR is negatively correlated with neuroendocrine differentiation in prostate cancer [24–26], however, in recent years, several studies have found co-expression of AR and NE markers in prostate cancer patients [25, 27, 28], and Su et al. [29] have found high expression of both AR and NE markers in a small subset of prostate cancer patients, especially among CRPC cases, suggesting AR might still exert its androgen response and anti-apoptotic effects in these patients which might be related to CRPC progression. Moreover, we also find that *PPIB-GAPDH* pair ranked 1^st^ among all communication combinations between cells from neuroendocrine subgroup and luminal subgroup in CRPC samples. *GAPDH* could enhance the transcriptional activity of *AR* in prostate cancer cells [18], and *PPIB* could regulate cell-cycle related genes including *CCDC74A* [30] which further promotes cell proliferation [31]. Taken together, this *PPIB/GAPDH/AR/CCDC74A* gene set might play an important role in regulating CRPC progression.

During tumor progression, various components from TMEs co-contribute to this process. CAF cells represent one of the most abundant components and play important roles in tumorigenesis, progression, metastasis and recurrence in prostate cancer [19]. CAF cells could also contribute to the development of CRPC, as reported in Eder et al. [32] and Kato et al. [33]. In our study, we found higher expression of FAP (CAF marker gene) in both fibroblast cells and CAF cells in CRPC samples compared to these in other samples, which is consistent with previous report [34]. Moreover, we also found higher expression of TGFB1 in CAF cells from CRPC samples compared to this in other samples. TGF-beta plays opposite roles in tumorigenesis in early stage of prostate cancer (inhibitor) and advanced stage of prostate cancer (promoter) [35], and an increased secretion of TGF-beta secretion in CAF cells might facilitate the CRPC development process.

Regarding immune features, we discovered a series of immunosuppressed phenotypes in CRPC samples such as enhanced M2 macrophage polarization process, enhanced Tcell exhaustion process, and increased number of regulatory B cells, suggesting that repressed immune system might facilitate CRPC progression. It is noteworthy that none of the immune-related cells are identified in GSE117403, which is consistent with its original publication [8]. This might be due to the filtering procedure during their single-cell library construction, and hence in this study we did not involve immune cells from normal samples, which might affect the comprehensiveness of our conclusions.

CRPC might arise from a small subset of cancerous cells [7], and due to the relatively small number of these cells, traditional bulk-sequencing results might not capture the minor expression changes of certain genes, which might also affect the performance of our algorithm, as most of the involved genes were identified through TCGA datasets. Moreover, compared to 6-gene panel, the 12-gene panel has only slightly better performance in separating HT samples with recurrence from those without recurrence, which might also be due to the limitation of bulk sequencing datasets. More datasets, especially scRNAseq datasets should be included to generate a more accurate algorithm. Lastly, the limited CRPC patient number is also a limiting factor of this study, and in the future, more patients should be involved in the validation process.

## Supplementary Material

Supplemental Material

## Data Availability

The datasets presented in this study can be found in online repositories. The names of the repository/repositories and accession number(s) can be found in the article/Supplementary Material.
