# Supplementary material for: A 12-gene panel in estimating hormone-treatment responses of castration-resistant prostate cancer patients generated using a combined analysis of bulk and single-cell sequencing data
Source: Ann Med. 2023 Sep 20;55(2):2260387. doi: 10.1080/07853890.2023.2260387 (PMC10512812; doi:10.1080/07853890.2023.2260387)
Supplement: Supplemental Material [file IANN_A_2260387_SM1990.zip › SFigures.docx]

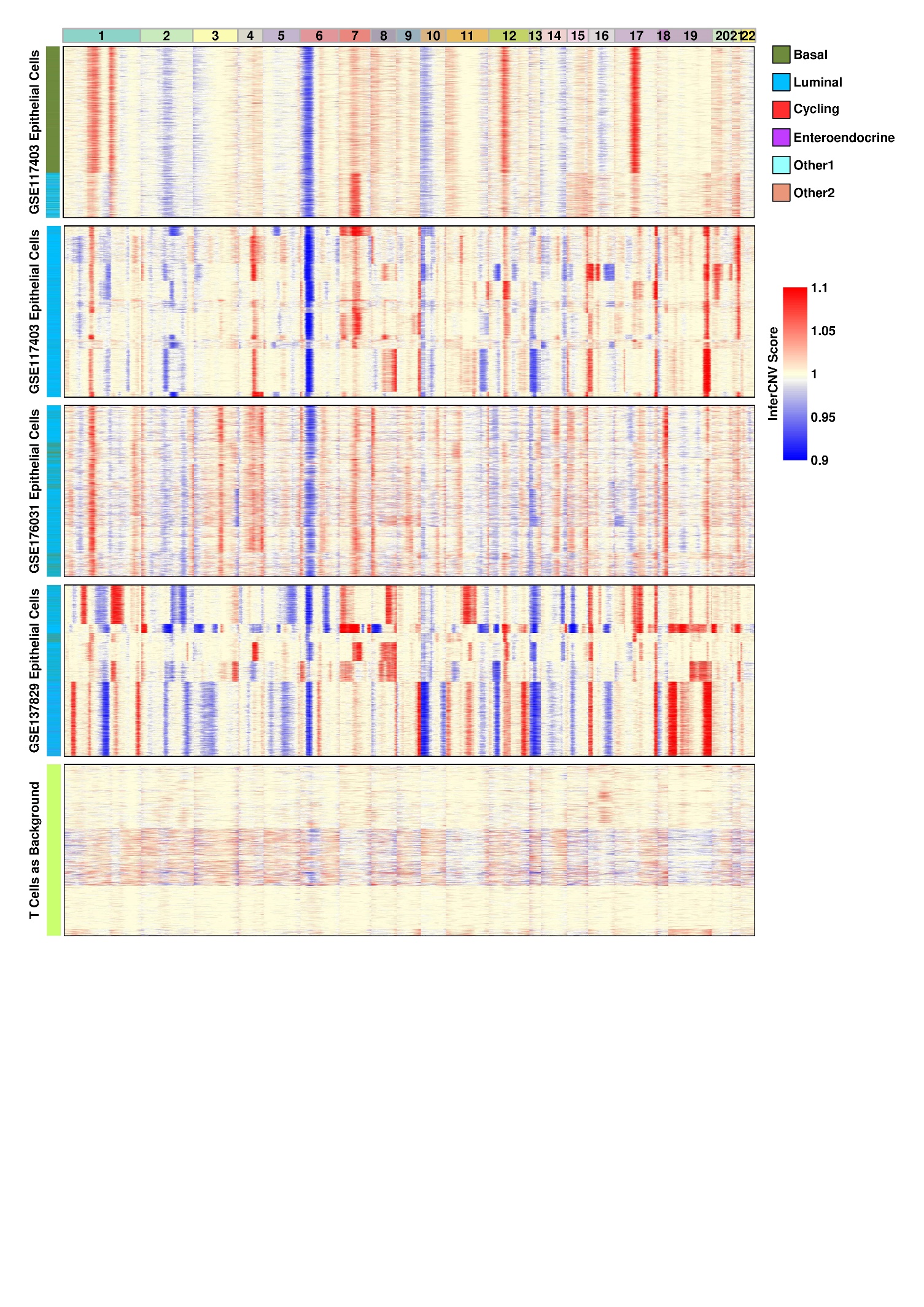


**Supplementary Figure 1.** InferCNV results of epithelial cells from four datasets. T cells were used as background
